# Nutrition in Critical Care

**DOI:** 10.1186/cc13957

**Published:** 2014-06-30

**Authors:** Carole Ichai

**Affiliations:** 1 Medicosurgical Intensive Care Unit, Saint Roch University Hospital, 5 rue Pierre Dévoluy, Nice 06000, France; 2 IRCAN Unit (Inserm U1081, CNRS UMR7284), Medical University of Nice, Nice 06000, France

## 

Faber P, Siervo M: *Nutrition in Critical Care*. New York: Cambridge University Press; 2014, 250pp, ISBN: 978-1-107-99901-7.

This interesting book represents a real challenge, giving the opportunity for various physicians to prescribe the most appropriate nutritional support in critically ill patients. The participation of 44 experts from a wide range of medical and paramedical subspecialities (physicians, dieticians, pharmacists) provides a complete approach to problems related to nutrition in these situations.

The first section includes general concepts and is divided into four chapters that underline the controversial methods used to assess nutrient requirements and the nutritional status. The following section covers the general management of nutrition in critical care (how to deliver nutrition, protocols and available commercial formulas). Gastrointestinal, metabolic and hormonal disturbances and their consequences on nutrition are clearly developed in the third section, followed by practical therapeutic guidelines or recommendations. The final section consists of 13 chapters dedicated to specific medicosurgical conditions (burn, neurological and digestive surgical patients, septic, immunocompromised and cancer patients), specific organ dysfunctions (kidney, liver, pancreatic, cardiac and pulmonary failures), and anorexic and obese patients.

The strength of this book is that each chapter gives practical recommendations or guidelines and concludes with major summary points. However, the major weakness is that the number of chapters is too high, causing redundant information to be present throughout the book. For example, each specific chapter begins with comparable reminders that are useless. This leads to an overlap and to repetitive information concerning the methods used to assess the energy balance, nutritional status, micronutrients and vitamin requirements. Moreover, each chapter dedicated to specific conditions repeats the same pathophysiological particularities, leading to duplicate information. A unique chapter summarising the real particularities of nutrition support for some specific conditions would have been easier and more practical. On the other hand, both of the chapters covering anorexia and obese patients remain justified.

In summary, *Nutrition in Critical Care* covers most of the important rules for nutritional support of critically ill patients. Despite numerous repetitions, the book will be useful for various medical and paramedical specialists (physicians, dieticians, and so forth) involved in the management of nutrition in critical care units.

## 

The licensee has exclusive rights to distribute this article, in any medium, for 12 months following its publication. After this time, the article is available under the terms of the Creative Commons Attribution License (http://creativecommons.org/licenses/by/4.0), which permits unrestricted use, distribution, and reproduction in any medium, provided the original work is properly cited. The Creative Commons Public Domain Dedication waiver (http://creativecommons.org/publicdomain/zero/1.0/) applies to the data made available in this article, unless otherwise stated.

## Competing interests

The author declares that she has no competing interests.

